# Perception of family relationships and depressive symptoms in older adults in Loja, Ecuador: an analytical cross-sectional study

**DOI:** 10.3389/fpubh.2026.1858931

**Published:** 2026-08-07

**Authors:** Tamara Rodríguez Quintana, Roxana Aimé Herrera Durañones, Daniela Cueva Vallejo, Serguei Iglesias Moré, Yordanis Arias Barthelemy, Justo Reinaldo Fabelo Roche

**Affiliations:** 1 Faculty of Health Sciences, Universidad Técnica Particular de Loja (UTPL), Loja, Ecuador; 2 Universidad de Ciencias Médicas de La Habana, Havana, Cuba; 3 Universidad de Ciencias Médicas de Santiago de Cuba, Santiago de Cuba, Havana, Cuba

**Keywords:** older adults, family relationships, depression, mental health, aging

## Abstract

**Background:**

Family relationships are an important psychosocial determinant of mental health in older adults. Their perception of family functioning may differ from that of other household members and influence the presence of depressive symptoms.

**Objective:**

To characterize older adults' perception of family relationships in Loja, Ecuador, and examine its association with depressive symptoms.

**Methods:**

An observational, analytical, cross-sectional study was conducted among 171 older adults and one cohabiting family member attending primary healthcare centers in Loja, Ecuador, between January and December 2024. Validated instruments assessed cognitive function (Mini-Mental State Examination), depressive symptoms (Geriatric Depression Scale-15), functional capacity (Barthel Index and Lawton and Brody Index), older adults' perception of family relationships (PRF), and family functioning reported by relatives (FF-SIL). Descriptive analyses, Spearman correlation, and multivariate binary logistic regression were performed. Crude and adjusted odds ratios (OR and aOR) with 95% confidence intervals (95% CI) were estimated, considering *p* < 0.05 as statistically significant.

**Results:**

Participants had a mean age of 75.1 years (SD = 8.3), and 70.2% were women. Most older adults (59.6%) perceived their family relationships as disharmonious, whereas 74.3% of family members reported adequate family functioning. Agreement between both perspectives was moderate (ρ = 0.315; *p* < 0.001). Older adults reporting disharmonious family relationships had significantly higher odds of depressive symptoms in the crude analysis (OR = 3.00; 95% CI: 1.40–6.42). This association remained significant after adjustment for sociodemographic variables (aOR = 2.41; 95% CI: 1.07–5.39) and after additional adjustment for clinical confounders (aOR = 2.35; 95% CI: 1.04–5.28). Per capita income below the unified basic salary was also independently associated with depressive symptoms (aOR = 3.67; 95% CI: 1.65–8.17).

**Conclusion:**

Older adults' perception of family relationships is independently associated with depressive symptoms, even after controlling for sociodemographic and clinical factors. These findings highlight the importance of incorporating older adults' own perspectives into the assessment of family dynamics and strengthening family-centered mental health promotion and prevention strategies within primary healthcare.

## Introduction

Population aging represents an increasing challenge for health systems and social structures. Approximately 10% of the global population is aged 65 years or older, and this proportion is projected to reach 16% by 2050 (1). In Latin America and the Caribbean, older adults account for nearly 10% of the population, with a faster growth rate compared to other regions (2). In Ecuador, around 9% of the population corresponds to older adults, with projections indicating a sustained increase in the coming decades (3). This demographic shift increases the demand for family support and health services tailored to the aging population.

In Latin American contexts, the family remains the primary informal care system. Family functioning, understood as a multidimensional construct that includes cohesion, communication, and adaptability, has been associated with both physical and mental health outcomes in older age (4, 5). Family dysfunction is linked to higher levels of psychosocial stress and a greater prevalence of depressive symptoms (6, 7).

Older adults' perceptions of their family relationships may differ from the evaluations made by other household members, particularly in emotional dimensions (8). Given that depression in older age has a multifactorial etiology and that psychosocial factors play a significant role, it is necessary to examine these dynamics from the perspective of older adults themselves. In Loja, no studies have simultaneously analyzed the perception of family relationships, family functioning, and their association with depressive symptoms. Therefore, the aim of this study was to characterize the perception of older adults in the city of Loja regarding family functioning and its association with depressive symptoms, during the period from January to December 2024.

## Materials and methods

### Study design

An observational, analytical, cross-sectional study was conducted using a quantitative design based on the objective measurement of variables and inferential analysis of their associations. The cross-sectional design allowed the simultaneous evaluation of the perception of family relationships, family functioning, and the presence of depressive symptoms in the study population.

### Setting and participants

The study was conducted at the primary healthcare level in an urban city in southern Ecuador. The study population consisted of older adults residing in the city and one cohabiting family member per participant. The total eligible population registered during the study period was included, resulting in a final sample of 171 dyads (older adult–family member).

Because the study included all eligible older adult–family member dyads registered at the participating primary healthcare centers during the study period, no a priori sample size calculation was performed. The final sample comprised 171 dyads and represented a census of the accessible population rather than a probability sample of all older adults residing in Loja. Consequently, the findings should be interpreted within the context of the participating healthcare centers and should not be considered representative of the entire older adult population of the city.

### Inclusion criteria

- Older adults affiliated with Zamora Huayco Health Center, Consacola Health Center, Carigán Health Center, La Pradera Health Center, and Health Centers No. 1 and 3 in Loja, who agreed to participate and signed informed consent.
- A family member living permanently with the older adult, who agreed to participate and signed informed consent.

### Exclusion criteria

- Older adults living alone or presenting cognitive impairment of sufficient severity to prevent understanding the study procedures or completing the instruments reliably were excluded. A MMSE score ≤ 26 alone was not considered an exclusion criterion
- Family members with cognitive impairment limiting their ability to respond to the instruments.

Justification for exclusion of older adults living alone: the study design required the simultaneous evaluation of the older adult's perception and the cohabiting family member's assessment (dyad). The absence of a permanently residing family member precluded the administration of the FF-SIL questionnaire and the contrast of perspectives within the household. It is recognized that this exclusion limits generalizability to older adults living alone, a subgroup with potentially higher depression risk. Therefore, the findings should be interpreted as applicable only to older adults living with at least one family member, since the study objective required comparison between both members of the dyad.

### Variables

The variables were classified as follows:

- Dependent variable: Presence of depressive symptoms in older adults, dichotomized as no depression (GDS-15 score ≤ 5) vs. probable or established depression (GDS-15 score ≥ 6).
- Independent variable: Older adults' perception of family relationships (PRF), categorized as disharmonious, slightly harmonious, harmonious, or very harmonious. Family functioning (FF-SIL), as reported by the cohabiting family member, was used for the correlation analysis and to compare perceptions within the dyad, but was not included as an explanatory variable in the logistic regression model.
- Potential confounding variables selected a priori based on previous literature: Age, sex, educational level (primary or less vs. secondary or higher), per capita income (< unified basic salary vs. ≥ unified basic salary), number of chronic diseases, functional capacity for basic activities of daily living (Barthel Index), and cognitive function (MMSE).

Sociodemographic variables included age, sex, ethnic self-identification, marital status, educational level, family type and size, and per capita income. Clinical variables included the presence of chronic diseases, neurosensory deficits, urinary incontinence, functional capacity for basic and instrumental activities of daily living, cognitive function, and depressive symptoms.

### Instruments

Cognitive function was evaluated using the Mini-Mental State Examination (MMSE), a 30-item screening tool assessing orientation, memory, attention, calculation, language, and visuospatial skills, with a maximum score of 30 points. A cutoff of ≤ 26 points was used to identify cognitive impairment, based on normative data generated for the Ecuadorian adult population (9).

Depressive symptoms were measured using the Geriatric Depression Scale by Yesavage in its 15-item abbreviated version (GDS-15), with dichotomous yes/no responses and a total score ranging from 0 to 15. The following cutoffs were applied: 0–5, no depression; 6–9, probable depression; 10–15, established depression. The GDS-15 has been validated in Spanish-speaking older adult populations and, specifically, in Ecuadorian older adults, where it showed adequate internal consistency (10).

Functional capacity was evaluated using the Barthel Index for basic activities of daily living (score 0–100), with higher scores indicating greater independence, and the Lawton and Brody scale for instrumental activities of daily living (Independent 8 points vs. dependent < 8 points). For the regression analysis, the Barthel Index was dichotomized as independent (100 points) and dependent (< 100 points). Both instruments are Spanish-adapted versions widely used in local clinical and research settings. Although formal psychometric validation studies in Ecuadorian older adults remain limited, both instruments are widely used in clinical practice and research throughout Latin America because of their robust international validation. The Barthel Index was selected for multivariable adjustment because it reflects dependence in basic activities of daily living, which is more closely associated with depressive symptoms than instrumental functioning. The Lawton and Brody Index was used only for descriptive purposes to avoid overparameterization of the regression model.

The perception of family relationships was evaluated using the Family Relationships Perception Test in Older Adults (PRF), a 30-item instrument with a 5-point Likert-type response scale (1 = almost never, 5 = almost always) and a total score ranging from 30 to 150. The following cutoffs were used: 146–150, very harmonious; 140–145, harmonious; 129–139, slightly harmonious; ≤ 128, disharmonious. The PRF has documented psychometric properties in other Latin American older-adult populations, but it has not yet been validated specifically in Ecuador. Internal consistency of the PRF was calculated for this sample using Cronbach's alpha (α = 0.9143), to complement the limited validation evidence available for this instrument in the Ecuadorian population.

Family functioning was evaluated using the FF-SIL Family Functioning Questionnaire, administered to the cohabiting family member. It comprises 14 items with a 5-point Likert scale, yielding a total score from 14 to 70, and classifies families as severely dysfunctional (14–28), dysfunctional (29–42), moderately functional (43–56), or functional (57–70). The FF-SIL evaluates seven specific dimensions: cohesion, harmony, communication, permeability, affectivity, roles, and adaptability. This instrument has been validated in Ecuadorian samples, although primarily in adolescent populations (11, 12). Although the available Ecuadorian validation has primarily involved adolescent populations, the FF-SIL was selected because of its conceptual consistency with the dimensions of family functioning evaluated in the present study and its extensive use in Latin American family research.

### Data collection procedure

Data collection was carried out through the in-person application of standardized structured instruments, composed exclusively of closed-ended questions and predefined response options. The evaluations were administered by the researchers in spaces that ensured privacy and comfort, with an approximate duration of 20–45 min per participant. All participants completed the assessment protocol, and no questionnaires were excluded because of incomplete responses attributable to interview duration. All instruments were applied following a standardized protocol under conditions of privacy.

### Statistical analysis

Data distribution was evaluated using the Shapiro–Wilk test for continuous variables, identifying a non-normal distribution. Consequently, Spearman's correlation coefficient was used to analyze the association between the perception of family relationships and family functioning, because both variables were ordinal and the assumption of normality was not met. Internal consistency of the psychometric instruments (GDS-15, PRF, and FF-SIL) was assessed using Cronbach's alpha coefficient. Values ≥0.70 were considered indicative of acceptable reliability.

To evaluate the independent association between older adults' perception of family relationships (PRF) and the presence of depressive symptoms, multivariate binary logistic regression was performed. Family functioning assessed with the FF-SIL questionnaire was analyzed separately using Spearman's correlation coefficient to examine its relationship with the older adults' perception of family relationships and was not included in the regression models because it represented the perspective of a different respondent within the dyad rather than an explanatory variable of the primary hypothesis. Potential confounding variables were selected a priori based on previous evidence regarding determinants of depressive symptoms in older adults. Three sequential logistic regression models were then constructed: (1) a crude model; (2) a model adjusted for sociodemographic variables (age, sex, educational level, and per capita income); and (3) a model additionally adjusted for clinical variables (number of chronic diseases, functional capacity for basic activities of daily living, and cognitive function). Additionally, the PRF total score was analyzed as a continuous variable (per 10-point decrease) in a complementary sensitivity analysis to evaluate the robustness of the observed association. Results were expressed as crude and adjusted odds ratios (OR and aOR) with 95% confidence intervals (95% CI). A *p*-value < 0.05 was considered statistically significant. All analyses were performed using IBM SPSS Statistics (version 29.0).

Model calibration was assessed using the Hosmer–Lemeshow goodness-of-fit test, and multicollinearity among independent variables was evaluated using variance inflation factors (VIF). No evidence of poor model fit or problematic multicollinearity was identified.

### Ethical considerations

The study was approved by the Human Research Ethics Committee of the Universidad Técnica Particular de Loja (CEISH-UTPL), under code 2023-11-INT-EO-RM-001 (version 2), which granted the corresponding ethical approval. All participants signed informed consent prior to inclusion. Confidentiality, anonymity, and exclusive use of the data for scientific purposes were guaranteed.

## Results

Table 1 presents the sociodemographic characteristics of the participating older adults. Female sex predominated (70.18%), the mean age was 75.1 years and marital status married/cohabiting was reported by 46.20% of participants. A total of 23.39% had primary education or less, and 51.46% reported per capita income ≥ the unified basic salary. A total of 77.19% presented at least one chronic disease. Internal consistency for the PRF in this sample was adequate/acceptable (Cronbach's α = 0.9143).

**Table 1:** Sociodemographic characteristics of participating older adults (*n* = 171).

Characteristic	*N*	%
**Sex**		
Female	120	70.18
Male	51	29.82
**Age (years)**	**75.1** **±8.3**	
**Educational level**		
Primary or less	40	23.39
Secondary or higher	131	76.61
**Marital status**		
Married/Cohabiting	79	46.20
Single/Widowed/Divorced	92	53.80
**Per capita income**		
≥ Unified basic salary	88	51.46
< Unified basic salary	83	48.54
**Chronic diseases**		
≥1 chronic disease	132	77.19
None	39	22.81

Age is presented as mean ± standard deviation (SD) for descriptive purposes despite its non-normal distribution. Categorical variables are presented as frequency and percentage.

Table 2 shows that 72.51% were independent in basic activities of daily living, while 34.50% presented partial or total dependence in instrumental activities. A total of 65.50% of participants had a normal cognitive screening result, whereas 34.50% screened positive for possible cognitive impairment using the MMSE cutoff of ≤ 26 points. Regarding mental health, 71.93% had no depressive symptoms, 18.13% had probable depression, and 9.94% established depression.

**Table 2:** Functional, cognitive, and affective status in older adults (*n* = 171).

Variable	*n*	%
**Basic activities of daily living (barthel index)**		
Independent (score = 100)	124	72.51
Dependent (score < 100)	47	27.49
**Instrumental activities of daily living (Lawton and Brody)**		
Independent (score = 8)	112	65.50
Dependent (score < 8)	59	34.50
**Cognitive function (MMSE)**		
Normal	112	65.50
Possible cognitive impairment	59	34.50
**Depressive symptoms (GDS-15)**		
No depression	123	71.93
Probable depression	31	18.13
Established depression	17	9.94

Possible cognitive impairment was defined as an MMSE score ≤ 26. This cutoff was used for cognitive screening and was not, by itself, an exclusion criterion.

Table 3 confirms that 59.65% of older adults perceived their family relationships as disharmonious and 35.67% as slightly harmonious. In contrast, 74.27% of family members classified their family as functional, 18.71% as moderately functional, 4.09% as dysfunctional, and 2.92% as severely dysfunctional.

**Table 3:** Perception of family relationships and family functioning (*n* = 171).

Variable	*n*	%
**Perception of family relationships (Older adult)**		
Very harmonious	4	2.34
Harmonious	4	2.34
Slightly harmonious	61	35.67
Disharmonious	102	59.65
**Family functioning (FF-SIL, family member)**		
Functional	127	74.27
Moderately functional	32	18.71
Dysfunctional	7	4.09
Severely dysfunctional	5	2.92

As shown in Table 4, in the crude logistic regression analysis, older adults who perceived their family relationships as disharmonious had three times higher odds of presenting depressive symptoms than those in the non-disharmonious group, which included slightly harmonious, harmonious, and very harmonious relationships (OR = 3.00; 95% CI: 1.40–6.42; *p* = 0.005). After adjustment for sociodemographic variables (age, sex, educational level, and per capita income), the association remained statistically significant (aOR = 2.41; 95% CI: 1.07–5.39; *p* = 0.033). In the fully adjusted model, which additionally included the number of chronic diseases, functional capacity for basic activities of daily living, and cognitive function, disharmonious family relationships remained independently associated with higher odds of depressive symptoms (aOR = 2.35; 95% CI: 1.04–5.28; *p* = 0.040). Additionally, a per capita income below the Unified Basic Salary was independently associated with higher odds of depressive symptoms (aOR = 3.67; 95% CI: 1.65–8.17; *p* = 0.001). No other covariate reached statistical significance in the fully adjusted modelIn Table 5, a positive and statistically significant correlation was observed between the perception of the older adult and the family functioning reported by the family member (ρ = 0.315; *p* < 0.001).

**Table 4:** Multivariable logistic regression analysis of factors associated with depressive symptoms among older adults.

Variable	Model 1 crude OR (95% CI)	*p*	Model 2 adjusted OR (95% CI)	*p*	Model 3 fully adjusted OR (95% CI)	*p*
**Perception of family relationships**						
Non-disharmonious	1.00	—	1.00	—	1.00	—
Disharmonious	3.00 (1.40–6.42)	0.005	2.41 (1.07–5.39)	0.033	2.35 (1.04–5.28)	0.040
**Per 10-point decrease in PRF score**	0.70 (0.58–0.84)	< 0.001	—	—	0.74 (0.61–0.91)	0.003
**Covariates included in Model 3**						
Age (years)	—	—	—	—	1.04 (0.99–1.09)	0.138
Female sex	—	—	—	—	1.35 (0.56–3.24)	0.497
Primary education or less	—	—	—	—	0.96 (0.41–2.28)	0.927
Income < Unified Basic Salary	—	—	—	—	3.67 (1.65–8.17)	0.001
Number of chronic diseases	—	—	—	—	0.96 (0.65–1.41)	0.839
Barthel dependence	—	—	—	—	1.76 (0.76–4.00)	0.189
Mild cognitive impairment	—	—	—	—	1.04 (0.47–2.33)	0.912

Model 1: crude model. Model 2: adjusted for age, sex, educational level, and per capita income. Model 3: additionally adjusted for number of chronic diseases, functional capacity (Barthel Index), and cognitive function (MMSE). Non-disharmonious relationships, used as the reference category, included slightly harmonious, harmonious, and very harmonious relationships. OR, odds ratio; CI, confidence interval; PRF, Perception of Family Relationships.

**Table 5:** Correlation between older adults' perception of family relationships and family functioning reported by family members.

Variables	spearman's rho	*p*
PRF score × FF-SIL score	0.315	< 0.001

## Discussion

Population aging constitutes one of the main contemporary demographic challenges, particularly in Latin America, where the sustained increase in the older population is associated with relevant structural and family transformations (1, 2, 13). In Ecuador, demographic projections confirm an accelerated transition toward an aging population (3, 14), reinforcing the need to understand psychosocial determinants influencing mental health in this age group.

The findings reveal a marked discrepancy between the older adults' perceptions and the family members' assessments of family functioning. While 59.6% of older adults perceived their family relationships as disharmonious, 74.3% of family members classified their families as functional. This divergence is consistent with the findings of Widmer et al. (15), who reported that older adults may experience and evaluate family conflicts primarily through relational and emotional dimensions, whereas other members of their family networks may prioritize organizational, economic, or instrumental aspects. These findings are also consistent with previous evidence regarding depressive symptoms and their associated psychosocial factors in older adults (16, 17). Similarly, Latin American studies have indicated that family functioning does not always fully reflect the subjective quality of affective bonds (4, 5, 18).

The positive correlation observed in this study between perception of family relationships and family functioning (ρ = 0.315; *p* < 0.001) is consistent with Zhou et al. (19), who highlight an interdependence between family cohesion and wellbeing in older age. However, as proposed by Guardiola (20), perception is a cognitive and emotional process mediated by previous experiences and individual expectations, explaining why two actors within the same family system may provide different evaluations.

The central finding of this study is that, after controlling for confounding variables known to be associated with depression, including age, sex, educational level, income, multimorbidity, functional capacity, and cognitive function, disharmonious family relationships remained significantly associated after adjustment with a 2.35-fold increase in the odds of depressive symptoms. This finding provides stronger evidence that the association between family relationships and depressive symptoms is independent of major sociodemographic and clinical determinants.

This finding aligns with Tibber et al. (14) and Widmer et al. (15), who report that family conflict increases the risk of depression in older adults. The World Health Organization recognizes that the social and family environment constitutes a key determinant of mental health in old age (21), and systematic reviews have shown that social support mediates the relationship between socioeconomic conditions and depressive symptoms (19).

The association between low income and depression (aOR = 3.67; 95% CI: 1.65–8.17) underscores the importance of structural determinants, as reported by Tibber et al. (14) and the WHO (21). Nevertheless, even after adjusting for socioeconomic conditions, the subjective quality of family relationships retained its predictive value. This aligns with the notion that the emotional impact of adverse family dynamics may not be fully buffered by material stability, particularly when the older adult perceives affective needs as unmet (15). Researchers such as Widmer et al. (15), in relation to family networks and quality of life, have shown that the presence of relational conflicts carries greater weight than the mere availability of instrumental support.

The relatively low prevalence of depression found (28.1%) could be linked to structural protective factors. Previous evidence shows that higher educational level and better economic conditions are associated with lower risk of depression in older adults (14, 22, 23). Likewise, income inequality has been related to poorer mental health at the population level (14), suggesting that relatively stable economic conditions may partially buffer the emotional impact of adverse family dynamics. However, even in contexts with adequate structural functionality, the presence of relationships perceived as disharmonious by older adults may affect their psychological wellbeing.

Interestingly, educational level, functional capacity, cognitive function, and multimorbidity were not independently associated with depressive symptoms after adjustment. Although these variables have been identified as relevant determinants in previous studies, the present findings suggest that older adults' perception of family relationships remained independently associated with depressive symptoms after adjustment, whereas the evaluated clinical characteristics did not. Nevertheless, the limited statistical power of the study warrants cautious interpretation.

Regarding the analysis of family functioning dimensions, the FF-SIL evaluation of the family member's perspective provides a structural view that evaluates cohesion, harmony, communication, permeability, affectivity, roles, and adaptability. While this instrument captures the organizational dimension of family life, it may not fully reflect the subjective emotional experience of the older adult. This reinforces the need for comprehensive clinical assessments that explicitly inquire about the older adult's perceived emotional quality of family relationships, rather than relying solely on proxy reports. These findings should also be interpreted within the Ecuadorian sociocultural context, where both nuclear and extended family structures coexist (24). Cultural norms regarding intergenerational support and emotional reciprocity may shape older adults' perceptions of family harmony, suggesting that future studies should explore whether these associations differ according to family structure and cultural context.

### Limitations

This study has some limitations that should be acknowledged. First, although the Barthel Index, the Lawton and Brody scale, and the PRF are widely used in geriatric clinical practice and recommended by national care protocols, such as the Ecuadorian Ministry of Public Health's Normas y Protocolos de Atención Integral de Salud de las y los Adultos Mayores (25), they have not been formally validated in the Ecuadorian population. Their use in this study relies on their established validity in other Spanish-speaking populations and their broad acceptance in regional clinical and research settings, including prior studies conducted in Ecuadorian samples (26).

For the PRF specifically, which has more limited psychometric documentation than the other instruments, internal consistency was calculated for the present sample (Cronbach's α = 0.9143) to provide additional, though not definitive, support for its reliability in this context. Future studies should prioritize the formal psychometric validation of these instruments including factor structure and construct validity in Ecuadorian older adults, to strengthen the local applicability of their cutoff points and classifications.

Second, the cross-sectional design prevents establishing temporality or causality between the analyzed variables and does not exclude reverse causation, as depressive symptoms may influence older adults' perceptions of their family relationships. Furthermore, the number of participants with depressive symptoms was limited relative to the number of parameters included in the fully adjusted model, which may have reduced the precision and stability of the estimates. Accordingly, the adjusted associations should be interpreted cautiously, particularly given the relatively wide confidence intervals. Although recent methodological literature suggests that logistic regression models may remain valid with fewer than the traditional 10 events per variable under appropriate conditions, the relatively limited number of outcome events may still have reduced the precision of some adjusted estimates. Therefore, the adjusted odds ratios should be interpreted cautiously. No missing data were observed for the variables included in the analyses, and therefore no imputation procedures were required. Additionally, the use of perception-based instruments may have been influenced by social desirability bias. The exclusion of older adults living alone restricts the generalizability of the findings to this subgroup, which may have a higher baseline risk of depression. Finally, the study included a census of the accessible population from selected primary healthcare centers rather than a probability sample of all older adults in Loja; therefore, the findings may not be generalizable to the entire older adult population of the city or to other urban and rural contexts.

## Conclusion

The evaluation of family dynamics in older age supports the integration of older adults' perceptions as a central component of clinical and psychosocial assessment. Structural assessment of family functioning may be insufficient if affective and relational components influencing emotional wellbeing are not considered.

These findings reinforce the need for comprehensive approaches at the primary healthcare level that incorporate the family dimension into mental health promotion and prevention strategies. They also highlight the relevance of future longitudinal research to further explore the mechanisms through which family interactions influence psychological health in old age, and to examine whether interventions aimed at improving family communication and emotional support can reduce the incidence of depressive symptoms in this population.

## Data Availability

The raw data supporting the conclusions of this article will be made available by the authors, without undue reservation.
